# Soluble epoxide hydrolase inhibitor promotes the healing of oral ulcers

**DOI:** 10.1016/j.clinsp.2023.100208

**Published:** 2023-05-04

**Authors:** Juanjuan Li, Zihan Wen, Yue Lou, Jili Chen, Lu Gao, Xiaojie Li, Fu Wang

**Affiliations:** a School of Stomatology, Dalian Medical University, Dalian, China; b The Affiliated Stomatological Hospital of Dalian Medical University School of Stomatology, Dalian, China; c Stomatological Hospital, Southern Medical University, Guangzhou, China; d Academician Laboratory of Immune and Oral Development & Regeneration, Dalian Medical University, Dalian, China

**Keywords:** Oral Ulcer, Ephx2 Protein, 1-Trifluoromethoxyphenyl-3-(1-propionylpiperidine-4-yl) Urea, Pain Measurement, Wound Healing

## Abstract

**Objective:**

Oral ulcers are a lesion in the oral mucosa that impacts chewing or drinking. Epoxyeicosatrienoic Acids (EETs) have enhanced angiogenic, regenerative, anti-inflammatory, and analgesic effects. The present study aims to evaluate the effects of 1-Trifluoromethoxyphenyl-3-(1-Propionylpiperidin-4-yl) Urea (TPPU), a soluble epoxide hydrolase inhibitor for increasing EETs level, on the healing of oral ulcers.

**Methods:**

The chemically-induced oral ulcers were established in Sprague Dawley rats. The ulcer area was treated with TPPU to evaluate the healing time and pain threshold of ulcers. The expression of angiogenesis and cell proliferation-related protein in the ulcer area was detected using immunohistochemical staining. The effects of TPPU on migration and angiogenesis capability were measured with scratch assay and tube formation.

**Results:**

Compared with the control group, TPPU promoted wound healing of oral ulcers with a shorter healing time, and raised pain thresholds. Immunohistochemical staining showed that TPPU increased the expression of angiogenesis and cell proliferation-related protein with reduced inflammatory cell infiltration in the ulcer area. TPPU enhanced cell migration and tube-forming potential *in vitro*.

**Conclusions:**

The present results support the potential of TPPU with multiple biological effects for the treatment of oral ulcers by targeting soluble epoxide hydrolase.

## Highlights

• The present results support the potential of TPPU to be a multi-functional therapeutic approach for oral ulcers by targeting soluble epoxide hydrolase.
• To provide a new idea for the treatment and prevention of clinical oral ulcers.

## Introduction

Oral ulcers are a common and frequently-occurring ailment in the mouth characterized by a loss of epithelium and underlying connective tissue, resulting in a crateriform appearance, which can occur on the lips, cheeks, tongue and gums.^1^ Most often occur in patients with recurrent aphthous ulcers.^2^ Patients may feel obvious pain when oral ulcers occur, especially when eating, drinking or brushing.

Although the pain and discomfort caused by oral ulcers will lessen in a few days and disappear in about two weeks without treatment, oral ulcers may also risk developing halitosis, chronic pharyngitis, irritable mood, headache, or other symptoms, causing great trouble to their daily life. However, since the pathogenesis hasn't been elucidated, there aren't effective measures to cure oral ulcers.^3^ Current clinical treatments are mainly anti-bacterial, anti-inflammation, immunomodulation, or anesthesia therapies.^4^ Therefore, there is a dire need for developing a therapeutic strategy that can accelerate the healing of oral ulcers with relief of pain, reduction of oral ulcer duration, and anti-inflammatory effects, which is more clinically meaningful.

Epoxyeicosatrienoic Acids (EETs) are a lipid medium produced by arachidonic acid metabolism. However, EETs are highly unstable *in vivo*, rapidly hydrolyzed by soluble Epoxide Hydrolase (sEH) into Dihydroxyeicosatrienoic Acids (DHETs).^5^ Inhibiting sEH can stabilize EETs levels and extend its half-life. Thereby, soluble Epoxide Hydrolase Inhibitor (sEHi) for increasing EETs levels demonstrates promise as a potential pharmaceutical agent for its multiple effects.^6^ Several studies have confirmed that increasing EETs directly or using sEHi to increase EETs levels by inhibiting its hydrolysis has anti-inflammatory, analgesic, accelerating wound healing, and pro-angiogenic effects.^7^,^8^

The EETs or using sEHi to increase EETs levels have been shown to reduce the inflammatory response and adhesion molecules expression, promote proliferation and the migration of endothelial cells for angiogenesis, and accelerate tissue growth, organ regeneration, and wound healing.^9^,^10^ sEHi can effectively treat gastrointestinal ulcers and colitis by inhibiting inflammatory cell infiltration and activation and enhancing epithelial cell defense.^11^,^12^ Whether these effects are also beneficial to the healing of oral ulcers requires further examination to expand their application.

Relieving pain to ease uncomfortable symptoms is one of the general measures for oral ulcer treatment. EETs or using sEHi to increase EETs levels have shown a peripheral anti-nociceptive effect that can block the Lipopolysaccharide (LPS)-induced inflammatory pain in rats, reduce neuropathic pain in a murine diabetic neuropathy model, reverse pain behavior in a mouse model of osteoarthritis.^13^, ^14^, ^15^ The potential of EETs for the treatment of central neuropathic pain has also been confirmed to attenuate nociception in a central poststroke pain model.^16^ The present previous study has demonstrated that 1-Trifluoromethoxyphenyl-3-(1-Propionylpiperidin-4-yl) Urea (TPPU), a sEHi, can contribute to sciatica relief in mice.^17^ However, whether TPPU is effective for the pain relief of oral ulcers also requires further examination.

In this study, the authors evaluated the effects of TPPU on cell migration and tube-forming potential *in vitro*, and confirmed that TPPU contributed to the healing of chemically-induced oral ulcers in rats by angiogenesis, reducing inflammation, relief of pain and reduction of oral ulcer duration.

## Materials and methods

### Oral ulcers model

Sprague Dawley (SD) rats (8 wk of age, male) were obtained from the Animal Experimental Center of Dalian Medical University. Rats were kept at room temperature 22°C, humidity 50%‒60%, and 12h light-dark cycle. All the experiments were approved by the Institutional Animal Care and Use Committee of Dalian Medical University (L2014035). This study compiled the Animal Research: Reporting *In Vivo* Experiments (ARRIVE) guidelines to minimize pain and suffering and to reduce the number of animals used.

**Fig. 1:**
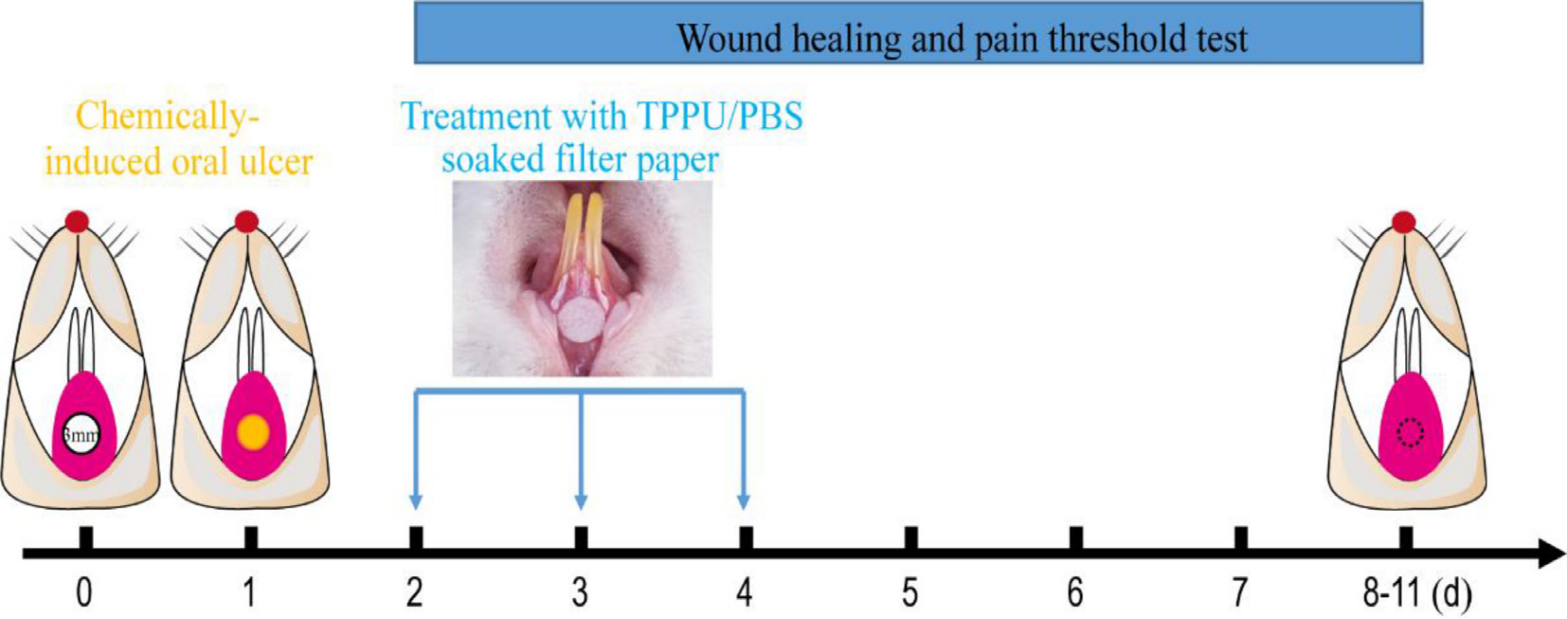
**Experimental protocol for chemically-induced oral ulcers and TPPU-treated.** A circular filter paper (diameter of 3 mm) soaked with 50% acetic acid was placed on the desiccative labial mucosa of the lower incisor alveolar bone of SD rats for 30s. After 48h, the oral ulcer areas were covered using the circular filter paper soaked with TPPU or PBS for 5 minutes daily for 3 consecutive days, and then the oral ulcer area and pain threshold was measured until the ulcer healing.

Oral ulcers were induced in rats using previously described methods.^18^ Briefly, Rats were anesthetized with Ketamine+xylazine (Ketamine, 60 mg/kg and xylazine, 5 mg/kg, intraperitoneal administration). The labial fornix region mucosa of the lower incisor was dried with a sterile cotton ball, then a circular filter paper (3 mm diameter) soaked in 50% acetic acid (Fuyu Fine Chemical, Tianjin, China) was placed in the labial mucosa of the lower incisor for the 30s (Fig. 1).

### TPPU treatment for SD rats

TPPU (Cayman Chemical Co. USA) stock solutions were prepared by dissolving TPPU in DMSO at the concentration of 12.5 mg/mL. The SD rats subjected to chemically-induced oral ulcers were randomly assigned into two groups: TPPU-treated groups (n = 5) and PBS-treated groups (n = 5, PBS pH = 7.4). The oral ulcer area was treated with a circular filter paper (3 mm diameter) soaked with TPPU (10 μM in PBS) or PBS for 3 days (5 minutes per day, Fig. 1). Normal SD rats were randomly selected as blank control and defined as NS group without any treatment (n = 5).

### The oral ulcer wound area tracing and pain measurement

Clinical evaluation for oral ulcers included appearance, healing time, and pain threshold assay. Successfully constructed oral ulcers were well-demarcated oral mucosal lesions with a central depression, yellow-gray pseudomembrane, and red margin (about 2.5‒3 mm. diameter). The days from the appearance of the ulcer to healing are defined as healing time. The wound bed appearance was recorded and photographed every day until the ulcer healing. The oral ulcer wound surface area was calculated using standardized digital imaging and the Image J software (National Institutes of Health, MD, USA) on days 3, 5, 7 and 9 after acetic acid treatment. Given that the oral ulcer may affect the diet of rats, the daily weight of SD rats was measured after acetic acid treatment, as a criterion for evaluating the effectiveness of treatment.

Pain sensitivity of the oral ulcer area was assessed using the manual Von Frey test every day until the ulcer healed. In brief, the threshold of mechanically evoked hyperalgesia-like behaviors was measured using von Frey filaments of varying forces (in grams) by an investigator blinded to the treatment group. Each filament was applied three times with a maximal duration of 2‒3s, and a 5s interval between each test. The pain threshold of normal SD rats without oral ulcers (NS group) served as a baseline.^18^

All examinations and evaluations of the ulcers were performed by two experienced dentists. The Kappa test for data from two evaluators is carried out to ensure consistency (Kappa values > 0.75 indicated that the assessment results were highly reproducible).

### Cell culture

Human Gingival Epithelial Cells (HGECs) were obtained for cell proliferation and wound healing assay, which were isolated and cultured as described previously.^19^ Briefly, primary HGECs were isolated from the gingiva tissue of patients with informed consent who underwent routine oral surgery at the Affiliated Stomatological Hospital of Dalian Medical University, Dalian. When reached 90% confluence, the cells were harvested and subcultured. The study was approved and followed the guidelines set by the Ethical Committee of the Affiliated Stomatological Hospital of Dalian Medical University (nº 2022001).

Human umbilical vein endothelial cells (HUVECs, ATCC, USA) were used for tube formation assay. HUVECs were cultured in endothelial cell medium (ECM, ScienCell, USA) with 5% fetal bovine serum (FBS, Gibco, Thermo Fisher Scientific, USA), 1% penicillin-streptomycin, and 1% endothelial cell growth supplement (ECGS, ScienCell, USA) at 37°C with 5% CO_2_.

### Histology, immunohistochemistry and immunofluorescence

The tissue on day-5 after 50% acetic acid treatment was isolated from the oral ulcer area in rats and was fixed in 4% Paraformaldehyde (PFA), and embedded in paraffin. 5 µm sections were prepared. The sections were stained with Hematoxylin and Eosin (H&E) to evaluate oral mucosal morphology and inflammatory cell infiltration. For immunohistochemistry, sections were incubated overnight with primary antibodies (rabbit polyclonal anti-CD31, rabbit polyclonal anti-Ki-67 and mouse monoclonal anti-VEGF, Abcam, Cambridge, MA, USA) at 4°C, then were incubated with secondary antibody for 30 minutes and horseradish labeled chain ovalbumin for 15 minutes (SP-9001, ZSGB-BIO, Beijing, China) at room temperature. Finally, sections were visualized using 3, 3-diaminobenzidine (DAB, ZSGB-BIO, Beijing, China), and counterstained with hematoxylin.^20^ Apoptosis levels were tested with the terminal deoxynucleotidyl transferase dUTP nick end labeling (TUNEL, BrightGreen Apoptosis Detection Kit, A112, Vazyme, Nanjing, China) according to the manufacturer's instructions. The percentage of cells with nuclear staining (Ki-67 or TUNEL staining) was calculated out of the total number of cells using Image-Pro Plus 6.0 software (Media Cybernetics, Rockville, MD, USA).

To detect the effect of TPPU on HGECs proliferation, HGECs were cultured in 96-well plates (1×10^5^/well) and treated with 10 μM TPPU (TPPU+ group) or without TPPU (TPPU- group) for 3 days, then the cells were fixed with 4% PFA and blocked in blocking buffer, and incubated with primary antibody (anti-Ki-67) in 4°C for overnight. Cells were then incubated with fluorescent secondary antibodies followed by nuclear DAPI staining.^20^

The images were taken under a fluorescence microscope (Olympus Corporation, Tokyo, Japan) and evaluated using Image-Pro Plus software.

### Tube formation assay

HUVECs were pretreated with TPPU (TPPU+ group) or without TPPU (TPPU- group) for 3d, The HUVECs cells were seeded into 96-well plates precoated with Matrigel (BD Biosciences, Franklin Lakes, NJ, USA) and incubated for 3h at 37°C. The formed capillary-like structures were fixed and observed under a light microscope (Olympus Corporation).^21^ The tube length and the number of nodes formed were quantified using Image-Pro Plus software.

### Wound healing assay

A total of 3×10^5^ HGECs per well were plated in 12-well plates overnight and treated with 10 μM TPPU (TPPU+ group) or without TPPU (TPPU- group) for 3 days. A single linear scratch was made with a 200 μL pipette tip. After gently washing the well twice, cells were cultured for 24h with TPPU or DMSO, then photographed. The width of the wound area was quantitatively evaluated using Image-Pro Plus software.^22^

### Statistical analysis

Data are presented as the mean ± SEM and were analyzed with SPSS13.0 software. An unpaired Student's *t*-test was used for comparison between different groups. Significance was defined as p < 0.05.

## Results

### TPPU accelerates wound healing and alleviates pain

**Fig. 2:**
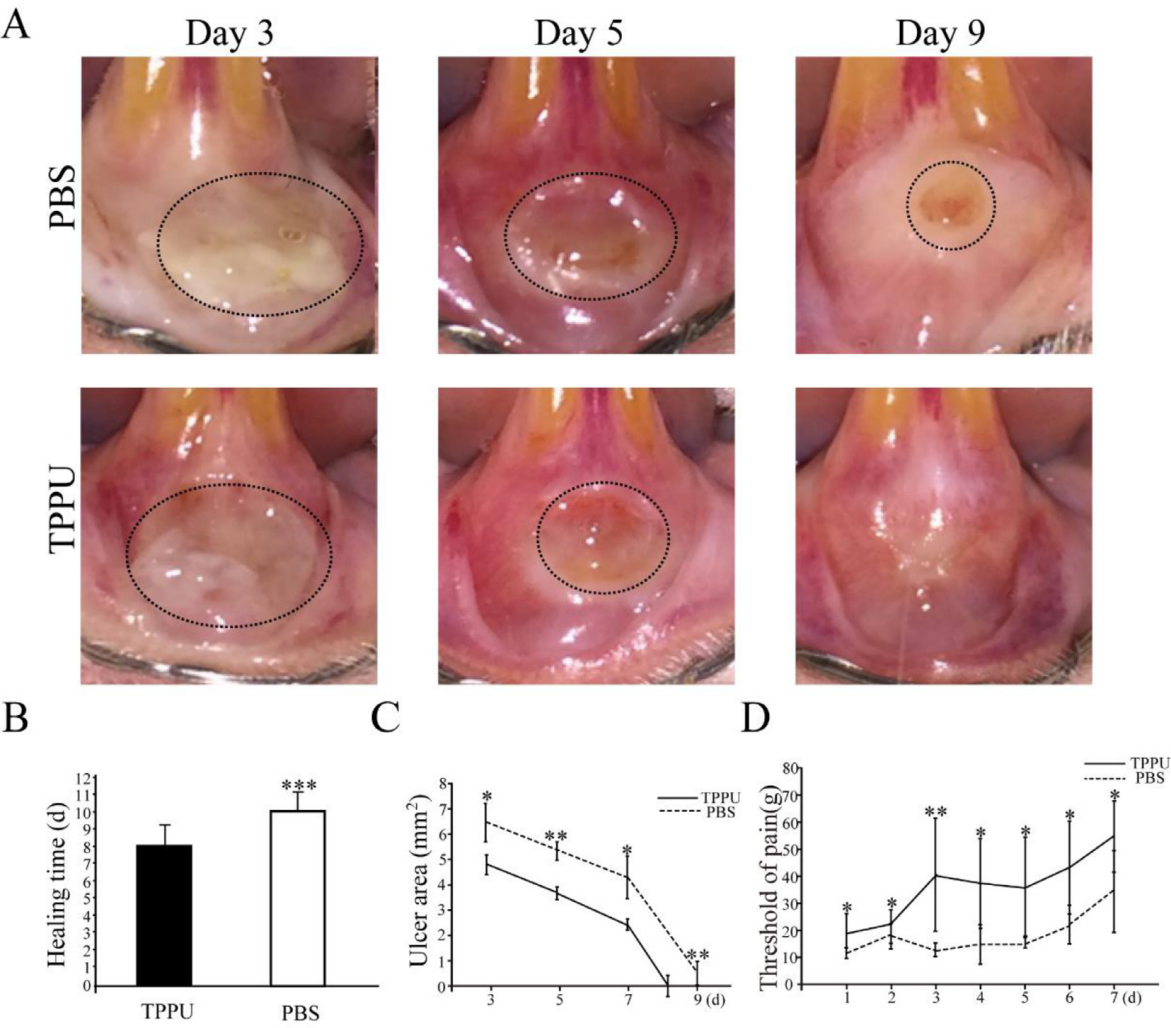
**Wound healing and pain threshold detection in oral ulcer area.** (A) Representative images of oral ulcers on days 3, 5 and 9 after acetic acid induction. (B) Comparison of oral ulcer healing time in TPPU-Treated groups (TPPU) and PBS-treated groups (PBS). (C) Quantification of oral ulcer area size on days 3, 5, 7 and 9 after acetic acid treatment in TPPU groups and PBS groups. (D) Comparison of pain threshold in oral ulcer area between TPPU groups and PBS groups. * p < 0.05, ** p < 0.01, *** p < 0.001.

TPPU-treated mice showed less weight loss and faster recovery than the PBS-treated group (data not shown). After 48 hours of acetic acid treatment, the appearance of the mucosa changed from pink to white. A uniform, shallow circular ulcer in the labial mucosa was clinically visible in each group. The oral ulcer bed was covered with a yellowish-gray pseudomembrane with a central depression and surrounding hyperemia swelling, and intense pain response to irritation (Fig. 2A).

From the 3^rd^ day after acetic acid treatment, the area of oral ulcers decreased gradually until they disappeared completely. While the appearance of wounds over time in the TPPU-treated group was significantly better than that in the PBS-treated group (Fig. 2A). The present results showed that the healing time of oral ulcers treated with TPPU was significantly shorter compared with the control group (8 days on the average in TPPU-treated group vs. 11 days on the average in PBS-treated group, (Fig. 2B, p < 0.001). Based on the above results, the authors further quantitatively compared the ulcer area size from 3 days to 9 days after acetic acid treatment (on days 3, 5, 7 and 9). Compared with the control group, the oral ulcer area was significantly reduced in the TPPU-treated group on days 3 (p < 0.05), 5 (p < 0.01) and 9 (p < 0.01), showing a more rapid healing process (Fig. 2C). Pain from oral ulcers can adversely affect eating and drinking. Therefore, the oral ulcer area was continuously measured by Von Frey to evaluate the pain tolerance of rats. The pain threshold of the oral ulcer in the TPPU-treated group was significantly higher than that of the control group (Fig. 2D, *p < 0.05, **p < 0.01), which indicated that TPPU can alleviate the pain sensitivity of the oral ulcer. The present results confirmed that TPPU could speed up oral ulcer healing and relieve pain.

### TPPU reduces inflammatory cell infiltration in the oral ulcer area

**Fig. 3:**
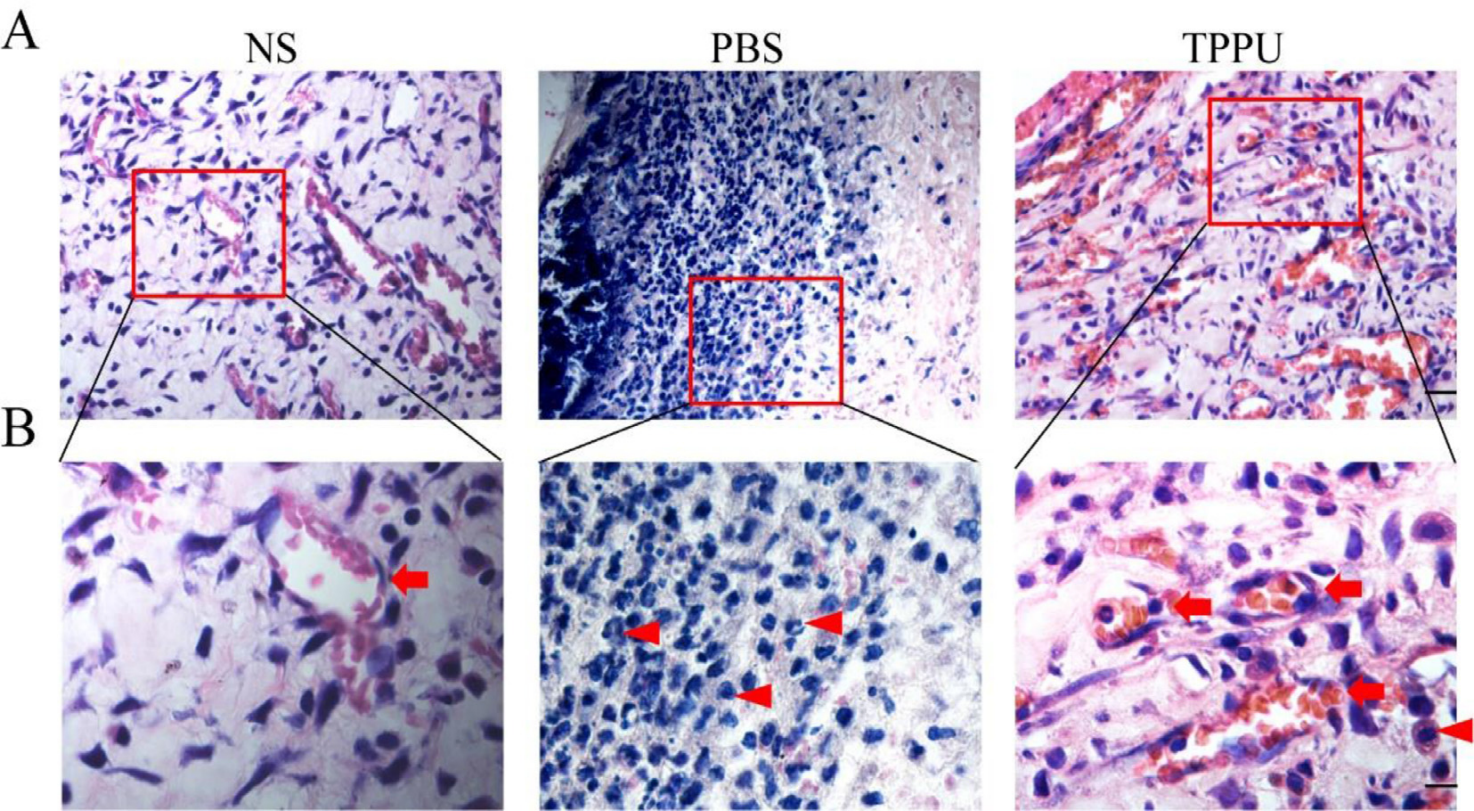
**TPPU reducing inflammatory cell infiltration in the oral ulcer area.** (A) Representative histological images of tissue from oral ulcer area in TPPU-Treated groups (TPPU) and PBS-Treated groups (PBS), the labial mucosa of the lower incisor alveolar bone from normal SD rats without any treatment (NS) was used as blank control. scale bar = 20 µm. (B) Magnified images of boxed areas in (A) showing the infiltrated inflammatory cells (red arrowhead) and angiogenesis (red arrow). scale bar = 10 µm.

In the special environment of the oral cavity, oral ulcers are often accompanied by inflammatory reactions, and anti-inflammatory also helps to speed up healing. To address this concern, the authors evaluated the anti-inflammation effects of TPPU. H&E analysis on day 5 revealed that the TPPU treatment attenuated the disruption of the epithelial layer, loss of basal membrane, and inflammatory cell infiltration in oral ulcer area, while there were many inflammatory cells, mostly lymphocytes and neutrophils infiltrated in PBS-treated oral ulcers (Fig. 3A, B). Meanwhile, the present results demonstrated that the tissue structure in the TPPU-treated group was consistent with that in the normal SD rats (NS group). In addition, lots of microvessels were identified in TPPU-treated group (Fig. 3B). The authors demonstrated that TPPU relieved inflammation severity and improved wound healing in oral ulcers. Results indicated that both inflammatory response and angiogenesis were involved in the healing of oral ulcers.

### TPPU promotes angiogenesis

**Fig. 4:**
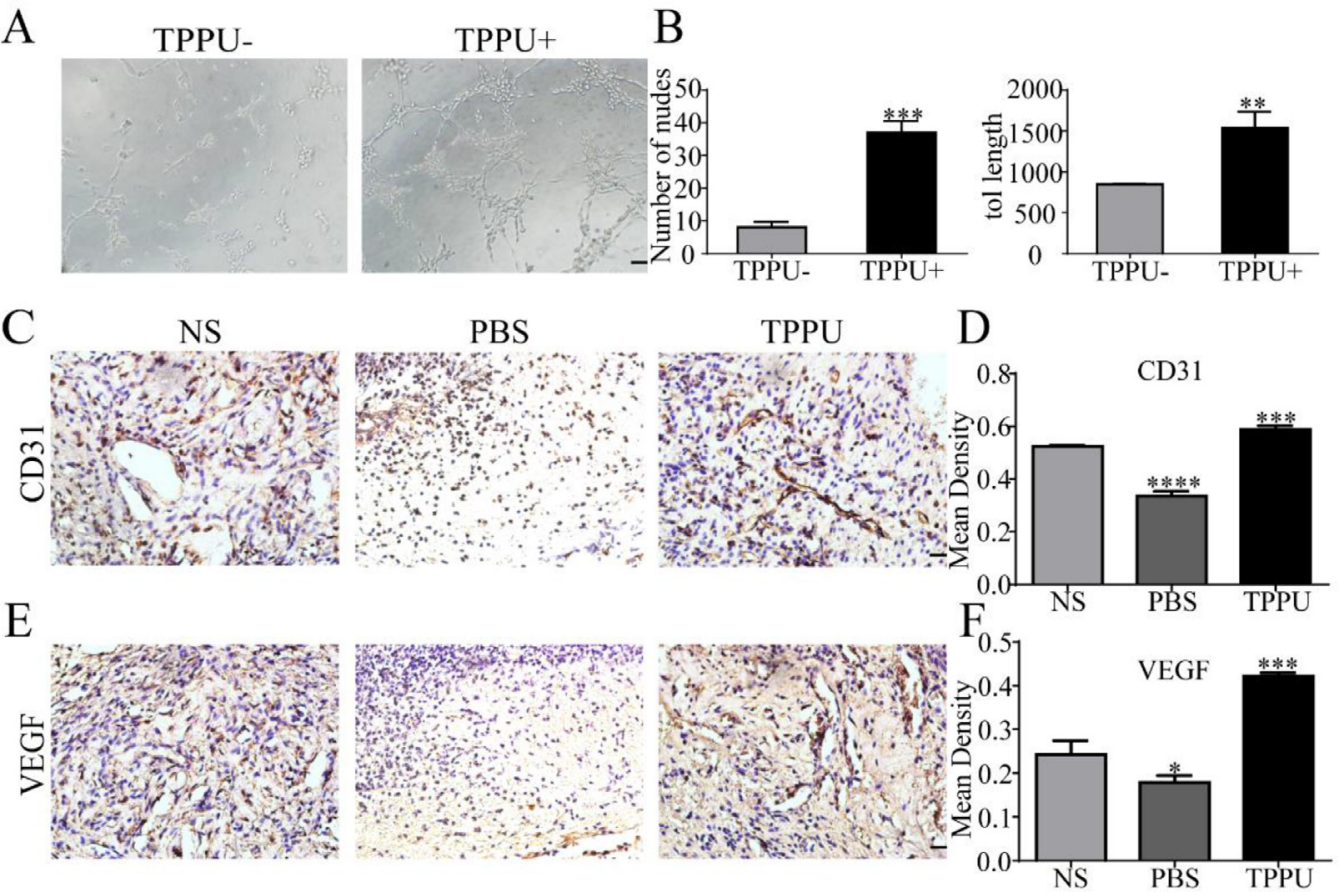
**TPPU promoting tube forming and angiogenesis.** (A) *In vitro* HUVECs (pretreated with/without TPPU) tube formation assay, scale bar = 200 μm. (B) Quantification of cumulative tube length and nodes for tube formation. (C) Immunohistochemical staining showing the CD31 expression in NS, TPPU-treated and PBS-treated groups, respectively, scale bar = 20 μm. (D) Quantification of CD31 expression. (E) Immunohistochemical staining showing the VEGF expression in NS, TPPU-treated and PBS-treated group, respectively, scale bar = 20 μm. (F) Quantification of VEGF expression. NS, normal SD rat without any treatment. * p < 0.05, ** p < 0.01, *** p < 0.001, **** p < 0.0001.

Angiogenesis can provide the oxygen and nutrients to growing tissue necessary for the metabolism of cells in the wound area and wound healing. *In vitro* tube formation test suggested that TPPU enhanced HUVECs tube formation (Fig. 4A) with more nodes number and tube length (Fig. 4B, p < 0.01). Next, the authors examined tissue in the oral ulcer area on day-5. CD31 and VEGF are well-studied markers of endothelial cells. To further examine the local vascularization of the oral ulcer, the authors measured the protein expression of CD31 (Fig. 4C, D) and VEGF (Fig. 4E, F) using immunohistochemistry staining. Compared with the normal SD (NS) group, TPPU-treated oral ulcers were significantly up-regulated (p < 0.001), while a remarkable down-regulation could be seen in the PBS-treated group (p < 0.05). These results implied that TPPU improving wound healing might be related to enhanced angiogenesis.

### TPPU inhibits cell apoptosis, enhances cell proliferation and migration

**Fig. 5:**
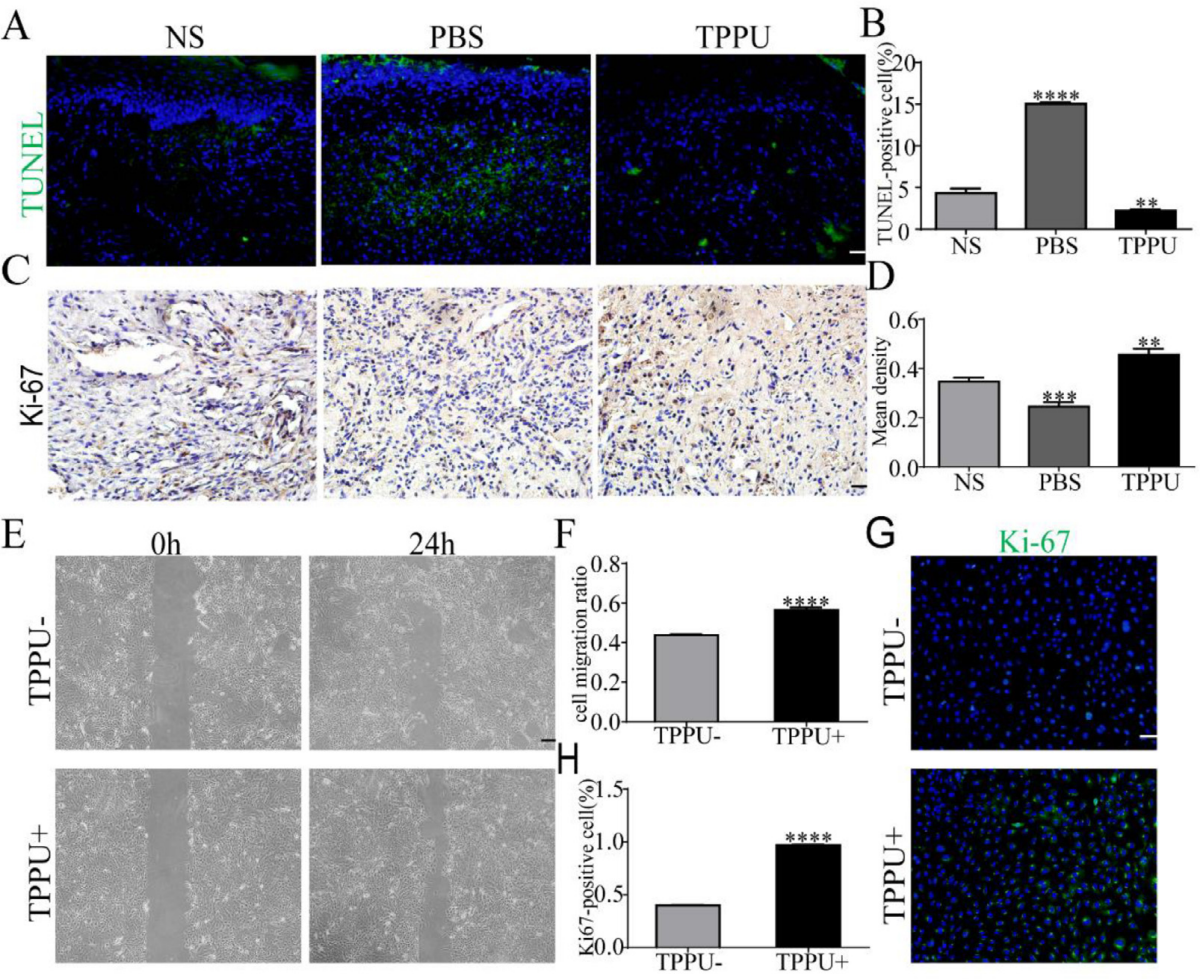
**TPPU inhibiting cell apoptosis and enhancing cell migration.** (A, B) Representative images and quantification of TUNEL staining of tissue from oral ulcer area, scale bar = 20 μm. (C, D) Immunohistochemical staining and quantification showing the expression of Ki-67 cells, scale bar = 20 μm. (E, F) Images and quantification of scratch wound assay of HGECs, scale bar = 200 μm. (G) Immunofluorescence staining showing the Ki-67+ HGECs treated with or without TPPU for 3 days, scale bar = 100 μm. (H) Quantification of the percentage of Ki-67+ HGECs in (G). ** p < 0.01, *** p < 0.001, **** p < 0.0001.

During the wound healing process, cell apoptosis and proliferation is the key to repair. To evaluate the effects of TPPU on wound healing, the authors examined the tissue from the oral ulcer area on day-5 using TUNEL staining and Ki-67 cell proliferation assays. TUNEL staining identified that TUNEL+ apoptosis cells were primarily represented in the center of oral ulcers in the PBS-treated group (Fig. 5A). Quantitative analysis showed that the apoptosis levels were significantly upregulated in the PBS-treated group (p *<* 0.0001) and were remarkably reduced in TPPU-treated group (p *<* 0.01) compared with the NS group (Fig. 5B). Ki-67, a proliferating cell-associated nuclear antigen expressed throughout the active cell cycle, was detected using immunohistochemistry. The present results showed there were more Ki-67+ cells in the TPPU-treated group (Fig. 5C, D; p *<* 0.01). Next, the authors evaluated the effect of TPPU on the migration and proliferation ability of HGECs. A scratch healing assay showed that TPPU accelerated wound closure (Fig. 5E, F; p *<* 0.0001). Cell proliferation assay demonstrated that the cells treated with TPPU (TPPU+) expressed higher cell proliferation marker Ki-67 than the cells treated without TPPU (TPPU-) (Fig. 5G, H; p *<* 0.0001).

## Discussion

The main treatment strategy for oral ulcers is usually palliative and supportive due to the complex pathogenesis of oral ulcers. Medications for oral ulcers include corticosteroids, non-steroidal anti-inflammatory/antiseptics, antibiotics, hyaluronic acid, topical anesthetics, and natural topicals.^23^ Further screening more effective medications with lower side effects to promote wound healing by reducing inflammation, relief of pain and reduction of oral ulcer duration is still a major challenge for the treatment of oral ulcers.^24^

Optimal wound healing is involved in multiple aspects: suitable angiogenesis, appropriate inflammation, cell proliferation, and migration to the wound site, prompting re-epithelialization and tissue remodeling. Angiogenesis provides nutrition and oxygen, growth factors secreted in the oral ulcer margin and base to promote epithelial cell migration and proliferation, which leads to final re-epithelialization, reconstruction, and maturation of the mucosal defect.^25^ EETs have been shown to possess multiple beneficial biological effects, however, the direct application of EETs is limited due to their rapid degradation by sEH.^5^ sEH inhibition is a promising therapeutic approach for addressing this issue by improving the endogenous activity of EETs. Our *in vitro* and *in vivo* results suggested that TPPU reduced inflammatory cell infiltration, promoted angiogenesis, enhanced cell proliferation and migration, and increased pain tolerance, which contributed to the healing of oral ulcers.

Angiogenesis is a key process during wound healing, providing the oxygen and nutrients necessary for the metabolism of cells in the wounded area.^26^ EETs as signaling molecules can promote multiple steps in angiogenesis, including migration and proliferation of endothelial cells, and formation of the vascular lumen.^27^ Experiments have shown that endogenous EETs accelerate angiogenesis and epithelialization in an ear wounds mouse model.^8^ The present experiments showed that TPPU greatly enhanced epithelial cell migration and proliferation, promoted angiogenesis and inhibited cell apoptosis.

Increasing evidence pointed out that EETs play anti-inflammatory effects by reducing inflammatory cytokine-induced endothelial cell adhesion molecule expression, inhibiting activation of the Nuclear Factor-κB (NFκB), inflammatory cytokines, and Cyclooxygenase-2 (COX-2) gene. Studies based on animal models have also confirmed that EETs/sEHi are effective in treating inflammatory diseases. The elevation of EETs could alleviate intestinal mucosal damage in ulcerative colitis.^28^ sEHi attenuates tobacco smoke-induced lung inflammation by reducing inflammation cells, including neutrophils, alveolar macrophages, and lymphocytes in the bronchoalveolar.^29^ 14,15-EET significantly reduces LPS-triggered Interleukin-1β (IL-1β) and Tumor Necrosis Factor-α (TNF-α) expression.^30^ The present results also confirmed the anti-inflammatory effect of TPPU in oral ulcers where the inflammatory cells in the TPPU-treated group were significantly reduced compared with the control group. It also indicated that inflammatory cells impact the growth and healing of oral ulcers. Since sEHi has an anti-inflammatory role,^31^ the local tissue damage in the oral ulcer area is reduced, and the healing time of the oral ulcers is shortened.

It has been confirmed that the gray area in the ventral aspect of the midbrain treated with 14, 15-EET in the thermal-induced painful tail-war model can produce a potent analgesic effect.^32^ In an osteoarthritis pain model, systemic administration of TPPU reverses established pain behavior in rats.^15^ In a type I diabetes rat model, sEHi elevates plasma and spinal Epoxy-Fatty Acids (EpFA) levels and reduces pain-related behavior.^33^ However, the underlying analgesic mechanisms remain poorly understood. Several studies point out that the analgesic effects of sEHi or EETs correlate with its anti-inflammatory processes. Inflammation can evoke pain by bioactive factors produced by local and migratory cells acting on sensory neurons.^34^ EETs can play an analgesic effect by reducing the production of algesic substances, including prostaglandins.^35^ A study suggests that the Steroidogenic Acute Regulatory Protein (StARD1) regulates EET-induced pain relief.^36^ It is hypothesized that sEHi enhances StARD1 expression, and subsequently induces the production of progesterone and other neurosteroids in the Central Nervous System (CNS) to exert analgesic effects. The present results also showed that TPPU increased pain tolerance, but the underlying mechanism needs further study. Since this analgesic effect can increase food intake, improve immunity, and weaken nervous tension, which undoubtedly contributes to oral ulcer healing.^2^

Due to the limitations of this experiment, the authors did not set up other treatment alternatives. It is necessary to further confirm the potential of TPPU in the treatment of oral ulcers through an in-depth comparison of TPPU and other alternatives such as topical corticosteroids or low-intensity lasers.

## Conclusions

The present results suggest that TPPU topical administration targeting sEH can be considered a potential therapeutic strategy for oral ulcers by exerting multiple biological effects of EETs, including shortening healing time, reducing inflammatory cell infiltration, promoting angiogenesis, enhancing cell migration and proliferation, which provides new ideas for the treatment and prevention of clinical oral ulcers. However, the medication method and mechanism need further exploration.

## Authors’ contributions

Fu Wang and Xiaojie Li designed the project. Juanjuan Li, and Zihan Wen performed animal experiments. Yue Lou and Jili Chen performed cytology experiments, data statistics and analysis. Lu Gao and Xiaojie Li kindly provided support during animal experiments. Juanjuan Li, Fu Wang wrote the manuscript. All the authors read and approved the final manuscript.

## Funding

This work was supported by the National Natural Science Foundation of China (nº 81771032); and the Natural Science Foundation of Liaoning Province (nº 2021-MS-293).

## Conflicts of interest

The authors declare no conflicts of interest.
